# A self-avoiding walk with neural delays as a model of fixational eye movements

**DOI:** 10.1038/s41598-017-13489-8

**Published:** 2017-10-11

**Authors:** Carl J. J. Herrmann, Ralf Metzler, Ralf Engbert

**Affiliations:** 1 Institute of Physics and Astronomy, University of Potsdam, Potsdam, D-14476 Germany; 2 Department of Psychology, University of Potsdam, Potsdam, D-14476 Germany

## Abstract

Fixational eye movements show scaling behaviour of the positional mean-squared displacement with a characteristic transition from persistence to antipersistence for increasing time-lag. These statistical patterns were found to be mainly shaped by microsaccades (fast, small-amplitude movements). However, our re-analysis of fixational eye-movement data provides evidence that the slow component (physiological drift) of the eyes exhibits scaling behaviour of the mean-squared displacement that varies across human participants. These results suggest that drift is a correlated movement that interacts with microsaccades. Moreover, on the long time scale, the mean-squared displacement of the drift shows oscillations, which is also present in the displacement auto-correlation function. This finding lends support to the presence of time-delayed feedback in the control of drift movements. Based on an earlier non-linear delayed feedback model of fixational eye movements, we propose and discuss different versions of a new model that combines a self-avoiding walk with time delay. As a result, we identify a model that reproduces oscillatory correlation functions, the transition from persistence to antipersistence, and microsaccades.

## Introduction

Eye movements are crucial for visual perception. Saccades shift regions of interest of a scene to the centre of the visual field, where high acuity vision is possible. Each saccade is followed by a period of fixation during which visual information is acquired. Even during these fixations, however, our eyes are never still, but rather perform miniature movements with movement amplitudes up to a degree of visual angle^1,2^. These involuntary and typically unconscious eye movements known as fixational eye movements (FEM) can be differentiated into three types of movement: drift, tremor, and microsaccades (MSs)^1,3^. Tremor is an aperiodic, wave-like motion with a frequency of about 90 Hz^3^ and amplitudes of about 0.02°^4^ (Note that tremor is not experimentally observable with video-based eye-tracking technology applied in this study). Drift, which is superimposed with tremor, is a slow, sometimes wave-like motion in the range of 0.02–0.1°^4^, with velocities of up to 0.5° per second^5^. Microsaccades (MSs) are rapid, ballistic movements of the eyes with amplitudes of about 0.5° and an approximate duration of 25 ms, interrupting drift and tremor about 1 to 2 times per second^3,6^.

Although we are not aware of these movements, they are nevertheless essential to maintain vision^1,3^. Several now classical experiments^7,8^ have shown that visual perception of a perfectly stabilised retinal image starts to fade rapidly due to the fast adaptation of retinal photoreceptor systems. Thus, there is an inherent tradeoff in visual fixation as the platform for visual perception: Fixational eye movements constantly move the retinal image across the photoreceptors to refresh their inputs and thereby to prevent visual fading^9^, while, at the same time, fixational eye movements maintain accurate fixation. The functional roles of the different movement types within the framework of this tradeoff have been discussed throughout the second half of the 20th century, yet they are not clarified with certainty. For example, it was found that MSs can be suppressed in high-acuity tasks^10–13^ and that in the case of reduced frequency or a complete absence of MSs, the drift component itself is capable of maintaining vision^9,14^ and accurate fixation^15–18^. Thus, it was argued that MSs serve no useful purpose^19^. However, there is also strong evidence that MSs serve both functions, i.e., counteracting visual fading^20^ and correcting drift-induced displacements^4,5,21^. There exists also a corrective role of microsaccades after blinks, and in the form of square-wave jerks^22–26^. Furthermore, recent neurophysiological studies reported a significant response of visual brain areas to MSs^2,3,27^, which indicates that MSs may indeed play an important role in vision. Beyond these discussions of rather basic functions, recent studies suggest that FEM enhance vision of fine spatial detail^28–32^ such as in high-acuity tasks, e.g., needle threading^33^. Several studies suggest that the enhancement of fine spatial vision is achieved by a temporal encoding of visual information mediated by precisely controlled retinal image motion^31,32,34^.

The debate on functional roles of FEM consequently leads to the question of their neuronal control, which has attracted increasing attention throughout the last years. Evidence has accumulated that MSs are centrally controlled in the superior colliculus^1,35–37^, a retinotopically organised structure in the brainstem which is also known to be responsible for the control of saccadic eye movements^38,39^. Drift movement has been considered to be determined by a neuronal control mechanism^19^ as well as merely by oculomotor noise^40^. A possible involvement of the superior colliculus in the control of drift movements is still under debate^41,42^. Research on the neuronal control of FEM, which is often based on neurophysiological data, can be supported by an approach which comes from statistical physics and computational modelling. The rather erratic nature of FEM motivates an interpretation and analyses within the framework of random walks and stochastic processes^43–46^. The concept of random walks, which can be traced back to Einstein’s work on Brownian motion^47^, nowadays attracts increasing attention in different scientific fields^48^, in particular in biology^49–51^.

A useful tool to characterise random walks is the mean squared displacement (MSD). In the case of Brownian motion, the MSD increases linearly with time lag *τ*, which is characteristic of random walks with uncorrelated increments. However, for many systems the MSD shows a non-linear dependence on the time lag which is an indicator of anomalous diffusion^52^. Such anomalous diffusion may have different physical sources^49,50,53^. In many cases, the MSD assumes the power-law form, i.e.,

1 $${\rm{MSD}}(\tau )\propto {\tau }^{\alpha },$$

generalising the linear dependence in the common case of Brownian motion, where the scaling exponent *α* indicates the type of diffusion^52^. For a definition of the discrete estimator of the MSD see Eq. (8) in the methods section. A scaling exponent *α* equal to unity refers to the case of classical Brownian motion or normal diffusion. If 1 < *α* < 2, the behaviour is referred to as superdiffusion, whereas for *α* > 2 the process is called hyperdiffusive. In the case of 0 < *α* < 1 we have a subdiffusive process. Mandelbrot and Van Ness^54^ coined the terms persistence and antipersistence for super- and subdiffusion, respectively, which we will adapt in the following to refer to the corresponding scaling behaviours of the MSD.

FEM are characterised by a fundamental scaling behaviour of the MSD first revealed by Engbert and Kliegl^43^. On a short time scale FEM are persistent whereas on a long time scale they are antipersistent. Engbert *et al*. estimated average scaling exponents of 1.4 and 0.69 for the short (2 ms to 20 ms) and long (100 ms to 400 ms) time scale, respectively. For the trajectories with removed MSs these exponents slightly decrease to 1.32 and increase to 0.97 for short and long time scales, respectively. These findings support that MSs serve both to counteract retinal adaptation by enhancing the persistence of the eye movements on the short time scale and maintaining accurate fixation by generating antipersistent behaviour on the long time scale. Furthermore, results of a study by Moshel *et al*.^55^ indicate that due to the influence of MSs the horizontal component of FEM are more persistent than the vertical component. Since these studies were based on data recorded during fixation of a single target, it is important to note that a new study by Amor *et al*.^56^ recorded FEM during a visual search task and observed no characteristic scaling behaviour of the MSD.

Motivated by the characteristic scaling behaviour of the MSD Mergenthaler *et al*.^45^ and Engbert *et al*.^44^ proposed computational models of FEM. First, Mergenthaler *et al*.^45^ modelled the activity of eye velocity related neurons in the brainstem using a first-order auto-regressive random walk with negative time-delayed feedback (NDF model). This approach follows the modelling of centre-of-pressure movements by Yao *et al*.^57^, which show a similar correlation structure of the MSD. The time delays of the horizontal and vertical movement, estimated by comparison of simulated and experimental data, are in good agreement with the knowledge of neural circuitries controlling eye movements^39,58^ and suggest separate control of both movement components. However, the NDF model lacks the integration of a mechanism for MS generation, which is worthwhile regarding the long standing debate on the neural control and functional roles of microsaccades^1–3^. In 2007, Engbert *et al*.^44^ proposed a fading self-avoiding walk (SAW, a random walk avoiding previously visited sites) which is constrained by a quadratic potential. The combination of a fading self-avoidance and the restoring force of a quadratic potential is a viable mechanism to model FEM, concerning the tradeoff between the refreshing of retinal input and an accurate fixation. Furthermore, the self-avoidance serves as a common generating principle for drift and MSs which is likely to be implemented in the superior colliculus.

Here we analysed the MSD of experimental FEM data with a special focus on inter-individual differences and the horizontal and vertical component. It turns out that the correlation structure of the MSD vigorously varies across the human observers, in particular, when MSs were removed from data. Furthermore, we found oscillating behaviour of the MSD at long times, which we investigated using the displacement auto-correlation function (DACF). These oscillations in the MSD and DACF support a central control of drift movements which may be governed by a time-delayed feedback. An analysis of simulated data from the two models indicates that the NDF model generates oscillations in the MSD and DACF while the SAW model does not. Therefore, we propose and discuss different implementations of a time delay in the SAW model in order to merge both models and reproduce the distinct correlations similar to that observed for the experimental data. Our results clearly show that a time delay has to be considered as a mechanism for drift control. However, the fact that the manifestation of oscillations significantly differs between the horizontal and vertical components and also across different human participants emphasises that the control of FEM is probably complex and may be influenced by different factors.

## Results

### Correlations in experimental data

**Figure 1:**
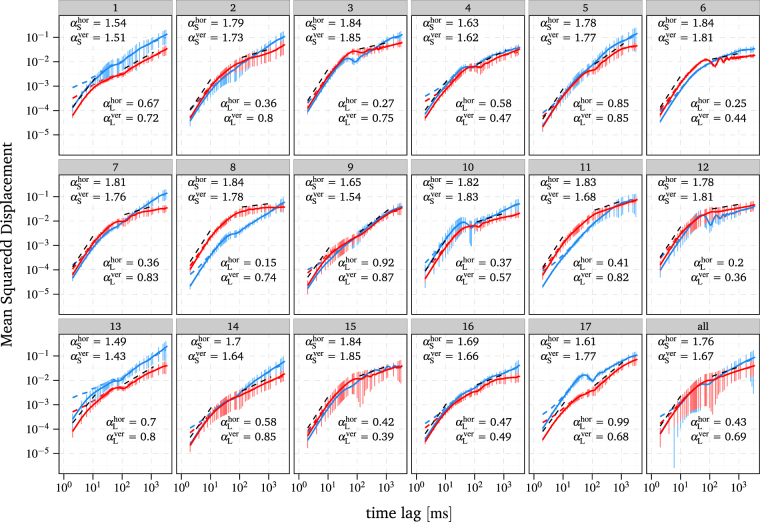
Log-log plot of the horizontal and vertical MSD of the smoothed experimental FEM data depicted in red and blue, respectively, with error bars indicating the standard deviation across the trials. The DACF of the raw, unsmoothed experimental FEM data is additionally depicted by the red and blue dashed lines. The estimated slopes of the short and long time scale for the smoothed data are denoted by *α*_S_ and *α*_L_, respectively. Furthermore, the estimated slopes for the horizontal component are illustrated as black dashed straight lines in the ranges used for the linear regression, i.e. 2–12 ms and 120–1200 ms for the short and long time scale, respectively.

The analysis of the MSD and DACF, see Eqs (8) and (9), is carried out for each of the 17 human participants to investigate for inter-individual differences. Thus, the MSD and DACF are averaged over all trials for each participant to obtain reliable estimates. Moreover, both the MSD and the DACF are calculated not only for the two dimensional trajectories, but also separately for the horizontal and vertical component of the movement. Because of the physiological separation into horizontal and vertical control loops, differences of the results between horizontal and vertical components will be highly informative on physiological mechanisms of the corresponding control loops. In the following, we present only plots of the components. Since our analyses could not identify differences between eyes, the following results were obtained from data of the left eye. Figure 1 shows the vertical and horizontal MSD for 17 individual probands as well as the average behaviour of all probands. Note that we applied a smoothing method to the experimental FEM data prior to all analyses, since the recorded trajectories do not represent the exact movements of the eye, due to measurement noise and the finite spatial resolution of the eye tracker. However, we additionally depict the results, obtained from the raw data, in order to show that the smoothing method does not have a strong qualitative influence on the data. For a detailed explanation of the smoothing method see the methods section.

#### Mean squared displacement

Our results for the scaling exponents of the MSD are in good agreement with those of Engbert *et al*.^43^ described in the introduction. For the scaling exponents of the two dimensional trajectories, averaged over all participants, we obtained numerical values of 1.75 and 0.58 for the long and short time scales, resp., which decrease to 1.67 at the short time scale and increase to 0.8 at the long time scale when MSs are removed from the trajectories, see Table 1. These scaling exponents for trajectories without MSs suggest that the drift movement is correlated at the short time scale and slightly antipersistent at the long time scale. However, the amount by which the scaling exponents change when MS are removed is found to vary across the participants at both the short and long time scale. Furthermore, the scaling exponents for removed MS show that drift can be almost uncorrelated as well as antipersistent at the long time scale, depending on the participant.

**Table 1:** Estimated scaling exponents of the MSD of the smoothed experimental FEM data for trajectories with and without MSs, marked in bold and plain text respectively.

Participant	Horizontal				Vertical				2D			
	${{\boldsymbol{\alpha }}}_{{\bf{S}}}^{{\bf{hor}}}$		${{\boldsymbol{\alpha }}}_{{\bf{L}}}^{{\bf{hor}}}$		${{\boldsymbol{\alpha }}}_{{\bf{S}}}^{{\bf{ver}}}$		${{\boldsymbol{\alpha }}}_{{\bf{L}}}^{{\bf{ver}}}$		${{\boldsymbol{\alpha }}}_{{\bf{S}}}^{{\bf{2D}}}$		${{\boldsymbol{\alpha }}}_{{\bf{L}}}^{{\bf{2D}}}$	
1	**1**.**54**	1.41	**0**.**67**	0.97	**1**.**51**	1.45	**0**.**72**	0.81	**1**.**85**	1.44	**0**.**54**	0.85
2	**1**.**82**	1.69	**0**.**37**	0.95	**1**.**83**	1.68	**0**.**57**	0.98	**1**.**83**	1.68	**0**.**50**	0.97
3	**1**.**83**	1.85	**0**.**41**	0.67	**1**.**68**	1.85	**0**.**82**	0.78	**1**.**79**	1.85	**0**.**59**	0.73
4	**1**.**78**	1.51	**0**.**20**	0.63	**1**.**81**	1.54	**0**.**36**	0.51	**1**.**79**	1.53	**0**.**26**	0.56
5	**1**.**49**	1.75	**0**.**70**	0.95	**1**.**43**	1.69	**0**.**80**	0.80	**1**.**45**	1.72	**0**.**78**	0.85
6	**1**.**70**	1.84	**0**.**58**	0.71	**1**.**64**	1.72	**0**.**85**	0.87	**1**.**67**	1.80	**0**.**76**	0.78
7	**1**.**84**	1.80	**0**.**42**	0.78	**1**.**85**	1.70	**0**.**39**	0.97	**1**.**85**	1.75	**0**.**40**	0.91
8	**1**.**69**	1.80	**0**.**47**	0.74	**1**.**66**	1.74	**0**.**49**	0.95	**1**.**67**	1.78	**0**.**48**	0.84
9	**1**.**61**	1.50	**0**.**99**	1.02	**1**.**77**	1.43	**0**.**68**	0.90	**1**.**73**	1.46	**0**.**78**	0.95
10	**1**.**79**	1.80	**0**.**36**	0.60	**1**.**73**	1.84	**0**.**80**	0.74	**1**.**76**	1.83	**0**.**61**	0.69
11	**1**.**84**	1.62	**0**.**27**	0.97	**1**.**85**	1.65	**0**.**75**	0.88	**1**.**85**	1.64	**0**.**53**	0.90
12	**1**.**63**	1.58	**0**.**58**	0.71	**1**.**62**	1.80	**0**.**47**	0.50	**1**.**62**	1.75	**0**.**52**	0.56
13	**1**.**78**	1.47	**0**.**85**	0.75	**1**.**77**	1.43	**0**.**85**	0.78	**1**.**77**	1.44	**0**.**85**	0.77
14	**1**.**84**	1.56	**0**.**25**	0.85	**1**.**81**	1.60	**0**.**44**	0.95	**1**.**83**	1.59	**0**.**37**	0.92
15	**1**.**81**	1.82	**0**.**36**	0.87	**1**.**76**	1.82	**0**.**83**	0.69	**1**.**79**	1.82	**0**.**65**	0.75
16	**1**.**84**	1.60	**0**.**15**	0.79	**1**.**78**	1.57	**0**.**74**	0.77	**1**.**83**	1.58	**0**.**31**	0.77
17	**1**.**65**	1.57	**0**.**92**	1.07	**1**.**54**	1.76	**0**.**87**	0.72	**1**.**61**	1.72	**0**.**89**	0.81
Mean	**1**.**73**	1.66	**0**.**50**	0.83	**1**.**71**	1.45	**0**.**67**	0.80	**1**.**75**	1.67	**0**.**58**	0.80
All participants	**1**.**76**	1.65	**0**.**43**	0.82	**1**.**67**	1.63	**0**.**69**	0.79	**1**.**75**	1.64	**0**.**57**	0.80

The linear regression for the estimation of the scaling exponents was applied in the range of 2–12 ms and 120–1200 ms for the short and long time scale, respectively.

**Figure 2:**
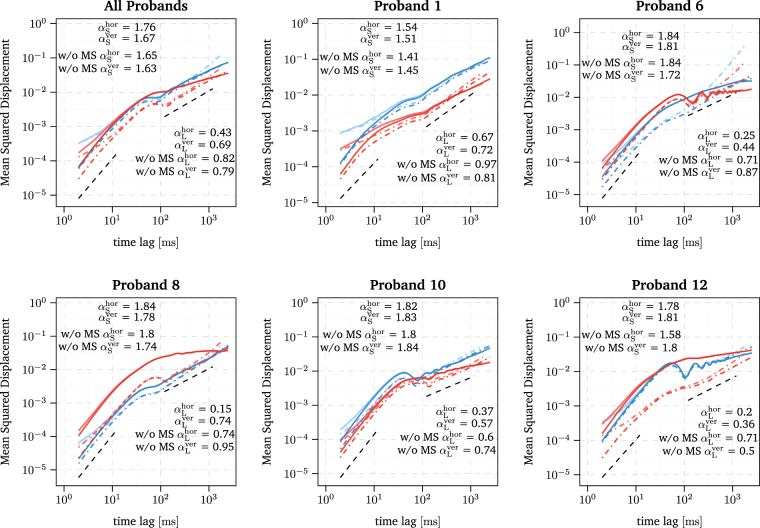
Log-log plot of the horizontal and vertical MSD of smoothed experimental FEM data depicted in red and blue, respectively. The solid and dot-dashed lines indicate the MSD of trajectories with and without MSs, respectively. The DACF of the raw experimental FEM data is additionally depicted with the same linetype but in a lighter shade of blue and red. The estimated slopes of the smoothed data for the short and long time scale are denoted by *α*_S_ and *α*_L_, respectively. Furthermore, the estimated slopes for the horizontal component without MSs are illustrated as black dashed straight lines in the ranges used for the linear regression, i.e. 2–12 ms and 120–1200 ms for the short and long time scale, respectively

This first main result is compatible with the view that human observers generate MSs and drift in different ways to achieve the goal of a prolonged and precise fixation of a point target. Furthermore, such a strategy must be based in an interaction of MSs and drift in order to achieve precise fixation. Note that our results do not confirm the findings of Moshel *et al*.^55^ that MSs increasingly enhance superdiffusion of the horizontal component at the short time scale, but rather suggest that MSs increasingly enhance antipersistence of the horizontal component at the long time scale and only a slight enhancement of superdiffusion at the short time scale. Note that in Figs 1 and 2 the MSD is depicted for raw and smoothed experimental FEM data. It shows that the applied smoothing induces a slight increase in the slope of the MSD at the short time scale. However, this does not represent a qualitative change in the data, but rather ensures a consistent scaling behaviour of the MSD at the long time scale. Below we show that the displacement autocorrelation function is fully insensitive to the smoothing.

Before we move on to different analyses, we would like to highlight the statistical behaviour of the MSD of the drift around the transition from short to long times (see Figs 1 and 2). First, for almost all participants, the MSD reaches a local maximum followed by a local minimum, and for some participants the MSD curve shows clear oscillations. Second, the behaviour differs in the horizontal and vertical components as well as between the participants. Third, this behaviour of the MSD is almost unaffected or even enhanced by removing MSs from the trajectories, see participant 8 in Fig. 2. The latter finding suggests that this correlation structure emerges due to drift movements. This oscillatory MSD function is an interesting feature, which might help to investigate the neuronal origin of drift and the principles of its control. An oscillating MSD is typical for systems with time-delayed feedback control^57,59,60^. Similar oscillations of the MSD, for example, although not as distinct as in our data, have been observed for the movement of the centre-of-pressure during quiet standing^57,60,61^.

#### Displacement auto-correlation function

**Figure 3:**
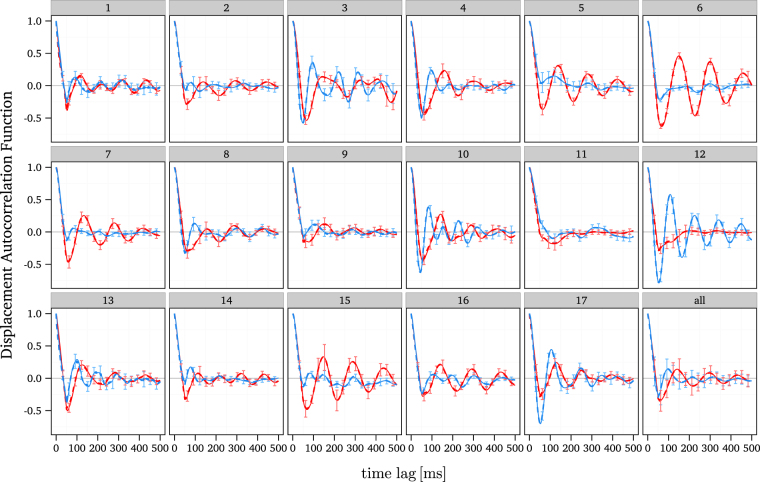
DACF of the horizontal and vertical component of the smoothed experimental FEM data depicted in red and blue, respectively, with error bars indicating the standard deviation across the trials. The DACF of the raw experimental FEM data is additionally depicted with dashed lines, which almost fully overlap with that of the smoothed data.

**Figure 4:**
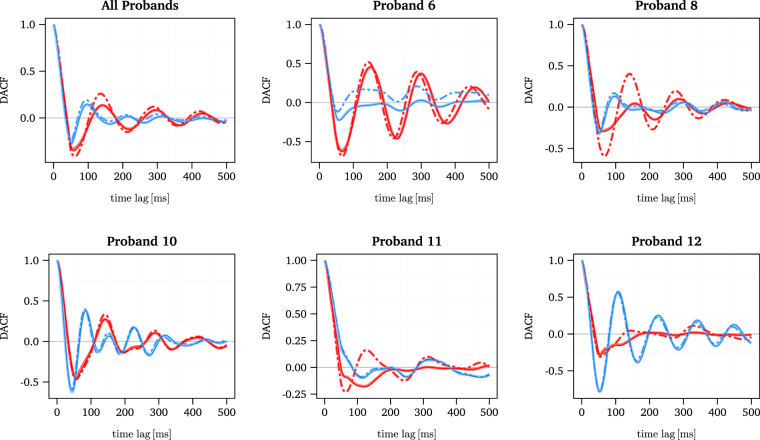
DACF of horizontal and vertical component of the smoothed experimental FEM data depicted in red and blue, respectively. The dot-dashed line denotes the DACF for trajectories in which MSs were removed. The DACF of the raw experimental FEM data is additionally depicted with the same linetype but a lighter shade of blue and red, which almost fully overlap with that of the smoothed data.

To further investigate these oscillations we calculated the displacement auto-correlation function (DACF). As can be seen from Fig. 3, the DACF exhibits a sharp decay crossing over to negative values followed by an oscillation between positive and negative values, for almost all participants. These oscillations obviously correspond to the oscillations observed in the MSD. Since the average of the DACF over all trials still shows oscillations, it can be concluded that they are not due to irregular, statistical fluctuations. As in the case of the MSD curve, removal of MSs from trajectories hardly affects or even enhances the oscillations in the DACF, see Fig. 4. This finding supports the interpretation that the drift component is a correlated movement which is generated by central neuronal control rather than resulting from oculomotor noise. Although the DACF averaged over all trials indicates a systematic structure of the oscillations, it becomes apparent that the precise behaviour differs between participants and between horizontal and vertical components of the movement, see Fig. 3. For example, data from participant 10 show oscillations in both components, whereas for participant 6 and 12, strong oscillations are limited to one component, while participant 11 exhibits only weak oscillations compared to other participants. Nevertheless, all participants have in common that the DACF of the horizontal component reaches the first maximum at a larger time lag (mean across probands: 135 ± 31 ms) than that of the vertical one (100 ± 33 ms), see Fig. 3, thus indicating that the oscillatory behaviour of horizontal drift movements tends towards lower frequencies. These results suggest that a possible implementation of a neuronal control mechanism of FEM varies between participants or may be influenced by other neuronal processes, but nevertheless facilitates a systematic difference between the horizontal and vertical component.

Oscillations of the MSD have been observed for centre-of-pressure movements during quiet standing^57,60,61^. Therefore, Yao *et al*.^57^ and Ohira *et al*.^60^ proposed different time-delayed stochastic systems to model these movements of the centre-of-pressure trajectory. Both models are capable of reproducing these oscillations for significantly large time delays. Further investigations of Ohira *et al*.^59,62^ on time-delayed stochastic systems showed that corresponding systems typically generate a damped oscillation of the auto-correlation function similar to that of the DACF observed in our data. These theoretical findings underline our previous observation that drift movements of the eyes may be controlled by a process which involves feedback that is time-delayed due to signal transmission times.

With respect to feedback control of drift movements, the most important questions for model building refer to (i) the level of central (brain) control and (ii) the type of signal that serves as a feedback. While there is evidence that the control of MSs involves the superior colliculus^35,36^, the functional role and control of drift movements is still under debate^41,42^. Estimating the time delays can help to draw conclusions on these questions. Theoretical studies^63–65^ investigating the behaviour of physiological control systems with a time delay *τ* indicate that the period *T* of the generated oscillations has a lower bound of two times the delay and may also have an upper bound of 4 times the delay,

2 $$2\tau  < T < 4\tau \mathrm{.}$$

Assuming that the periods of oscillations in FEM are approximately the same as the periods of the DACF, i.e., 100 to 200 ms, Eq. (2), we obtain an estimate of the neural delay time of about 25 to 100 ms. Thus, an involvement of cortical areas in a possible feedback loop can be ruled out, since this would imply delays of at least one order of magnitude higher^66^. However, the range of the time delay is compatible with an involvement of the superior colliculus. It takes about 20 ms for the generation of an eye movement by a motor command sent from the superior colliculus^39^ and about 40 ms for the retinal signal to travel to the superior colliculus^67^, thus adding up to a lower bound of 60 ms for the time delay for control loop which involves visual signals. However, eye movements can also be controlled by efference copies^68,69^, i.e., copies of movement-producing neuronal signals, which operate without delays.

In summary, our second main result is that the drift movement generates oscillations in the MSD and DACF, which lends supports to the view that drift is controlled by a time-delayed feedback loop. The time scale of these oscillations is compatible with an involvement of the superior colliculus.

### Correlations in simulated data

We now turn to the analysis of correlations in the data generated by the non-linear delayed feedback (NDF) model^45^ and the fading self-avoiding walk (SAW) model^44^ of fixational eye movements. Both models are motivated by the characteristic scaling behaviour of the MSD which they reproduce. Therefore, we will investigate which of the two models reproduce the oscillations in the MSD and DACF found for the experimental data. We simulated 30 trials with 10,000 steps, which corresponds to the size of the experimental data set. We applied the same algorithms for preprocessing (smoothing) and averaged the MSD and DACF over the (simulated) trials, see methods section. In the following, we give a brief description of both models. For a more detailed description and the mathematical definitions see the methods section and the corresponding references^44^ and^45^.

The NDF model proposed by Mergenthaler *et al*.^45^ follows the modelling of centre-of-pressure trajectories during quiet standing^57^ using a time-delayed stochastic differential equation. Neurophysiologically, the model is derived from the fact that the activity of oculomotor neurons in the brainstem is determined by the sum of the activities of tonic units and excitatory burst neurons, which are related to the eye position and eye velocity, resp. As the change of eye position during FEM is negligible, the activity of the tonic units is modelled as the sum of a noise term and the activity of the excitatory burst neurons *w*_*i*_. The activity *w*_*i*_ is determined by the sum of a noise term, representing the baseline activity, an auto-regressive term considering the eye’s inertia and a non-linear, negative feedback with time delay. The negative feedback aims to stabilise the FEM and generates, due to the time delay, an antipersistent behaviour on the long time scale^45^.

As the NDF model lacks a mechanism for MS generation, Engbert *et al*.^44^ proposed the SAW model, which incorporates a common generating mechanism for drift and MSs. In the SAW model, the next step of the walker depends on the values of the sum of a self-generated activation *h* and a quadratic potential *u* at the four neighbouring sites of his current position. Basically, the procedure is as follows. As the walker visits a lattice site (*i*, *j*), he increases the activation *h*_*i*,*j*_ by one unit, in the following referred to as *activation setting*. Next, the sum of the potential and the activation of the four neighbouring sites is read out to determine the next step, since the walker will always visit the site with the lowest value of this sum. In the following, we will refer to this as *state readout*. Next, the determined step is executed and the procedure is repeated. After each step, the activation is globally decreased by multiplying it with the relaxation rate *ε*. In the case that two or more neighbouring sites show the same value, one of them is chosen with equal probability. If the walker happens to visit a lattice site which exhibits an activation above the critical value *h*_*c*_, an MS is triggered towards the global minimum of the sum of the activation field, the quadratic potential and the microsaccadic potential. The microsaccadic potential thereby ensures that MSs occur predominantly in the horizontal and vertical direction. Neurophysiologically, the lattice represents the superior colliculus, a retinotopically organised structure located in the brainstem which is involved in microsaccade and saccade generation^35,38,39^. The restoring force of the quadratic potential may likely be implemented by tonic units in the brainstem, which are related to the eye position. Note that for the current SAW model we did not take the logarithmic scaling of the superior colliculus^70^ into account, but used a linearly scaled quadratic lattice, see the methods section. However, in future variants of the SAW model a logarithmic scaling should be considered, as well.

**Figure 5:**
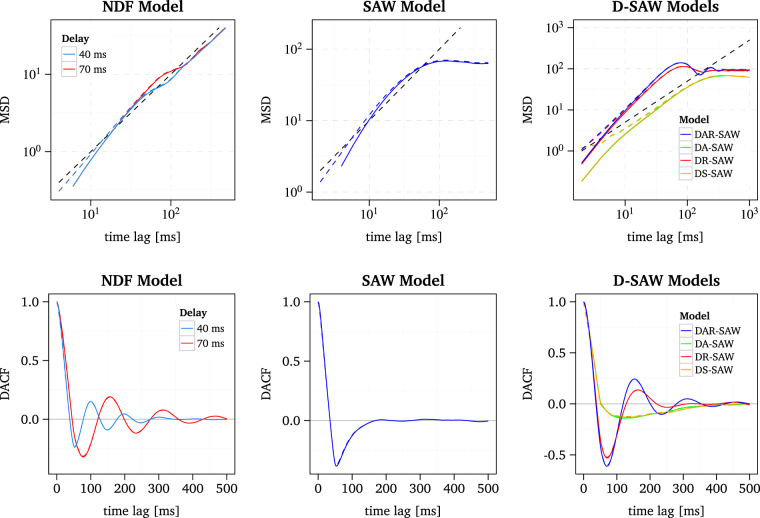
MSD and DACF of the NDF model, the SAW model and the different implementations of a delay in the SAW model. Results from raw and smoothed simulated data are depicted as solid and dashed lines, respectively. For a key to the abbreviations of the different D-SAWs see Table 2. Note that the NDF model was simulated with a delay of 40 ms and 70 ms, corresponding to the optimal delay of the vertical and horizontal component, respectively, regarding the match of simulated and experimental data^45^. The black dashed line in the log-log plot of the MSD indicates a slope of one.

As can be seen from Fig. 5, the NDF model reproduces oscillations in the DACF. This could have been expected from the general structure of the NDF model that is similar to the time-delayed random walks studied by Ohira *et al*.^59,62^. In their study of the NDF model, Mergenthaler and Engbert^45^ estimated different time delays for the horizontal and vertical eye movement component, i.e., 70 ms and 40 ms, resp. Here we used these two different delays for our simulations of the NDF model. As a result, for the larger delay of 70 ms, the DACF reaches the first maximum at a larger time lag compared to the delay of 40 ms (associated to the vertical component)^45^. These results are similar to the behaviour of the horizontal and vertical DACFs we observed for the experimental data of all participants, see Fig. 3.

In contrast to the NDF model, the SAW model does not reproduce the oscillations in the MSD and DACF. The DACF decreases to negative values and then asymptotically converges to zero. Such a dependence of the DACF on the time lag may be explained by the interplay of the quadratic potential and the self-generated activation field in association with the principle of the fading self-avoidance. Due to the fading, self-avoidance depends on the time scale, so that the walker maintains his current direction of movement at the short time scale (persistence), however, after many time steps, the walker reaches lattice sites with high activation or high potential values and is pushed back to its initial position (antipersistence).

In conclusion, our analyses of experimental data favour the NDF over the SAW model based on the MSD and DACF results. However, the SAW model has the main advantage that it implements a mechanism for microsaccade generation. Thus, it is desirable to merge both modelling approaches, by implementing time-delayed feedback in the SAW. In the following, we discuss different implementations of time delay in the SAW on the basis of the correlation analyses of simulated data.

#### Time-delayed SAWs

We start with a summary of properties of the delayed random-walk model investigated by Ohira *et al*.^62^ to get an idea of the delay that is necessary to generate the desired oscillations in the DACF. The random walk is realised in one dimension with one unit step per time unit. The probability *p*(Δ*x* = ±1|*x*(*t*)) to take a step to the left or right at time *t* depends on the position *x*(*t*), so that the probabilities to take a step to the right for *x*(*t*) < 0 (i.e., Δ*x* = +1) and to take a step to the left for *x* > 0 (Δ*x* = −1) are greater than 0.5. Thus, without delay, the random walker is attracted to the origin *x* = 0. With a time delay *τ* the step probability *p*(Δ*x* = ±1|*x*(*t* − *τ*)) depends on the time-delayed position *x*(*t* − *τ*) and the origin becomes less attractive as it might happen that the stepping probability depends on a position in the negative half-line, while the current position of the walker is in the positive half-line, and vice versa. With sufficiently large time delays this effect naturally generates an oscillatory behaviour of the random walker.

In the SAW model, the quadratic potential *u*(*i*, *j*) plays a role comparable to that of the step probability *p*(Δ*x* = ±1|*x*(*t*)), since it acts as a restoring force which drives the walker towards the origin. The self-generated activation field, however, acts like a local repulsive force which pushes the walker away from recently visited lattice sites. Due to the statistical self-avoidance, the walker can reach lattice sites with high values of the potential *u*, from where he is then driven back towards the origin. This interaction of potential and activation field, mediates the statistical self-avoidance and generates the negative correlations of the DACF in the SAW, see Fig. 5, but generates uncorrelated behaviour on the long time-scale. Based on these considerations, it appears promising to implement a time delay with respect to the position for the state readout in the SAW model to reproduce the experimentally observed oscillations of the DACF. Alternatively, we also considered a time-delayed position for activation setting and a time delay of the full activation state *h*. In the following, we give a brief description of the different implementations of D-SAW model variants, which we considered in our analyses (Table 2).

**Table 2:** Overview of the different types of delays we implemented in the SAW.

Model	Activation state	Position for	
		Activation setting	State readout
DS-SAW	delayed	—	—
DR-SAW	—	—	delayed
DA-SAW	—	delayed	—
DAR-SAW	—	delayed	delayed

The dash denotes that no delay is applied. Here, *activation state* refers to the activation field the walker generates as he moves, *activation setting* to the local increase of the activation the walker executes at the visited lattice sites and *state readout* to the evaluation of the next step on the basis of the four neighbouring values of the sum of the activation *h* and the potential *u*.

**DASAW**  implements activation setting using time-delayed position. At time *t*, the activation is increased by one unit at lattice site *x*(*t* − *τ*), which was visited by the walker at time *t* − *τ*, while state readout is executed at the current position of the walker. In the simulation, the decrease of activation is paused for the first *τ* steps of the burn-in phase (see Methods).

**DR-SAW**  uses the time-delayed position for the state readout. Thus, the current activation state is read out at position *x*(*t* − *τ*), visited by the walker at time *t* − *τ*. As for DS-SAW, the walker moves randomly with isotropic stepping probability for the first *τ* iterations of the burn-in.

**DAR-SAW**  denotes a model version with both DA-SAW and DR-SAW. Thus, the time-delayed position *x*(*t* − *τ*) is used for activation setting and state readout. As for the latter implementations the walker moves randomly with isotropic step probability for the first *τ* iterations of the burn-in. Furthermore, the decay of the activation field is interrupted for the first *τ* steps of the burn-in.

**DS-SAW**  implements readout of time-delayed activation states. This means that the state readout is executed at the current position using the activation values at time *t* − *τ*. For the first *τ* steps of the burn-in the walker moves randomly with isotropic step probability.

Note that in the case of DR-SAW and DAR-SAW we had to adjust the threshold for MS triggering *h*_*c*_ in the range of 15 to 20, compared to *h*_*c*_ = 7.9 in the original SAW model, in order to obtain MS rate similar to the corresponding rate in the SAW model and in the experimental data. Moreover, we observed that for the DR- and DAR-SAW models the walker often gets trapped in confined trails, in particular for low delays. We solved this problem by adding a low-amplitude noise in the read-out position of the DR- and DAR-SAW, i.e., with a certain probability a neighbouring lattice site of the actual position is chosen for the readout and the activation setting, respectively. To this end a Gaussian random number, $\delta \sim {\mathscr{N}}\,(\mu =0,\,{\sigma }^{2}=0.09)$, which is rounded to the nearest integer, is added to the actual position **x**_*i*_,

3 $${{\bf{x}}}_{i,{\rm{error}}}=({x}_{i}+{\rm{round}}({\delta }_{x}),{y}_{i}+{\rm{round}}({\delta }_{y})),\quad {\rm{with}}\quad {\delta }_{x},{\delta }_{y}\sim {\mathscr{N}}\mathrm{(0,0.09),}$$

to obtain the erroneous position **x**_*i*,error_, which is used for the readout and activation setting, respectively. From a neurophysiological perspective this solution is reasonable as information in the nervous system is encoded in firing rates which are naturally affected by noise. Note that we also implemented this approach for the DS- and DA-SAW model, although we did not encounter a problem with the walker getting trapped in confined trails for these models, to ensure that the performance of the D-SAW models can be compared with respect to the different delay types.

In Fig. 5 the MSD and DACF are depicted for the different implementations of the D-SAWs. It turns out that the DA-SAW as well as the DS-SAW do not exhibit oscillations in the DACF. Furthermore, it is apparent that both models show nearly the same time evolution of the MSD and DACF. This could be explained by the similarity of the readout of the time-delayed activation states associated with the current activation setting and the readout of the current activation state associated with the delayed activation setting. In both models the influence of the activation is, to some extent, decoupled from the current position of the walker. The walker rather likely visits lattice sites with high activation due to which he is repelled in some direction, but also lattice sites with low activation where he is repelled less strongly. Moreover, the interaction of the fading self-avoidance and the restoring force of the potential is also decoupled, which we consider to be primarily responsible for the strong negative correlation in the DACF of the SAW, see Fig. 5, which might explain the low negative displacement autocorrelation, as well as the observation of a slope of the MSD close to one at the short time scale (i.e., almost uncorrelated movements).

The two other models, namely, the DR-SAW and the DAR-SAW, exhibit oscillations of the DACF similar to that in the experimental data. In the case of the DAR-SAW, the MSD exhibits oscillations at the long time scale, which were also observed for some participants in the experimental data. Both models have in common that they implement time-delayed positions for the state readout. In addition to the DR-SAW, the DAR-SAW makes also use of the time-delayed position for activation setting. Thus, the simulations confirm our expectation that a time delay of the position for the state readout could generate the experimentally observed oscillatory behaviour. This finding represents our third main result, which points out that the DAR-SAW and DR-SAW models are superior to the NDF model and the original SAW model.

**Figure 6:**
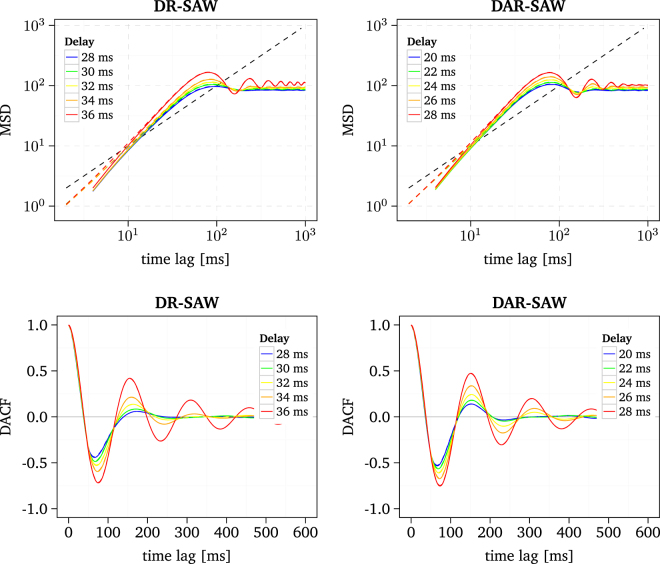
MSD and DACF of the DR-SAW and the DAR-SAW for different delays. Results from raw and smoothed simulated data are depicted as solid and dashed lines, respectively. For a key to the abbreviations of the different D-SAWs see Table 2.

For further investigations on the DR-SAW and the DAR-SAW we simulated the models for different time delays *τ*. Generally, it turns out that both models are highly sensitive to the numerical value of the time delay, see Fig. 6. Furthermore, the amplitude of the DACF appears to exhibit a non-linear dependence on the time delay. For example, the DR-SAW generates a large increase of the oscillation in the MSD and DACF for a delay change from 34 ms to 36 ms. Additionally, with increasing delay, the persistence, i.e., the slope at the short time scale of the MSD, is enhanced. However, the increase of the time delay does not result in an increase of the period of the DACF, which was found for the NDF model. These patterns of behaviour of the MSD and DACF can be explained as follows. Due to the time-delayed position for the state readout, it is likely that the walker visits lattice sites with high activation values (i.e., recently visited sites) and also lattice sites with high values of the potential. Thus, the walker is repelled more strongly, enhancing the persistence at the short time scale. In addition the time-delayed position for the state readout in association with the quadratic potential generates an oscillating autocorrelation in the long time range similar to the step probability dependence of the time-delayed position in the random walk model proposed by Ohira *et al*.^62^. The enhanced persistence may also be caused by the increase of MSs which are triggered when the walker reaches lattice sites with high activation values. This can be regulated by increasing the threshold *h*_*c*_. However, the number of triggered MSs exhibits strong fluctuations across the simulated trials. Thus, a refined analysis and and further adjustments of the two D-SAW models will ultimately be needed.

Finally, we discuss the problem of how to create different behaviour of the horizontal and vertical components in the D-SAW type of models. Since an implementation of different time delays for the horizontal and vertical component, like in the NDF model, is implausible in the D-SAW and, furthermore, such a modification could not result in different periods of the DACF, the question arises how the different properties of the components can be realised in the D-SAW models. Having in mind the considerations of the role of the potential in the SAW and its similarity to the step probability in the model of Ohira *et al*.^62^, different values of the slope parameter *λ* for the horizontal and vertical direction of the quadratic potential,

4 $$u(i,j)=L({\lambda }_{{\rm{hor}}}\,{(\frac{i-{i}_{0}}{{i}_{0}})}^{2}+{\lambda }_{{\rm{ver}}}\,{(\frac{j-{j}_{0}}{{j}_{0}})}^{2})$$

should generate a difference between the components. As the horizontal component exhibits a larger period than the vertical component, we decided to set the slope parameters to *λ*_hor_ = 0.9 and *λ*_ver_ = 1.1.

**Figure 7:**
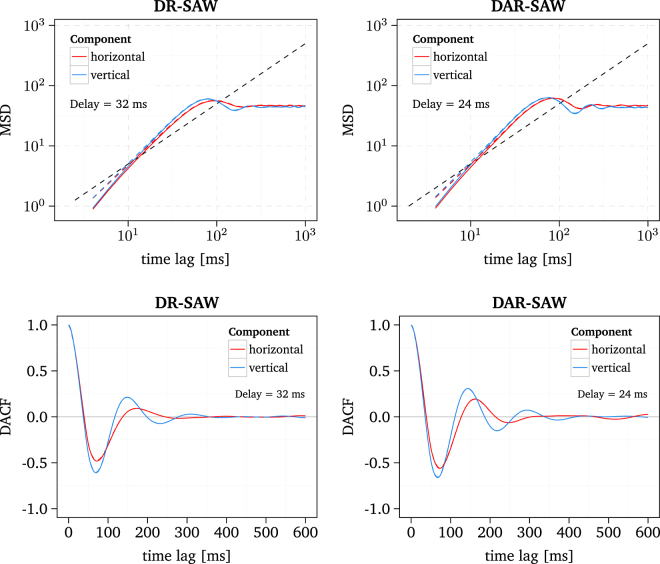
MSD and DACF of the DR-SAW and the DAR-SAW with a different slope parameter *λ* of the quadratic potential *u* for the horizontal and vertical direction, *λ*_hor_ = 0.9 and *λ*_ver_ = 1.1. Results from raw and smoothed simulated data are depicted as solid and dashed lines, respectively. For a key to the abbreviations of the different D-SAWs see Table 2.

The effect of different values for *λ*_hor_ and *λ*_ver_ on the behaviour of the DACF is obvious, see Fig. 7. The lag of the first maximum of the horizontal component is larger than that of the vertical component. Moreover, the DACF of the horizontal component decays faster than that of the vertical component which is in good agreement with the behaviour of the DACF of most participants. This suggests that the difference in the time course of the horizontal and vertical DACF could arise due to an anisotropic control of fixation deviation. Furthermore, such a mechanism could explain the finding that for some participants the DACF exhibits strong oscillations for one component while oscillations are not observed for the other component. Here, a future goal should be to investigate if there is a relation between the horizontal and vertical fixation deviation and the lag of the first maximum of the oscillating DACF.

## Discussion

We analysed the mean squared displacement and the displacement autocorrelation function in fixational eye movements and investigated the role of neural delays in theoretical models for the explanation of experimental data.

Our first main result of the correlation analyses of experimental FEM data by means of the MSD and DACF revealed that the drift movement is not uncorrelated but rather exhibits a scaling behaviour of the MSD which varies across participants, suggesting that drift is under central control. It is found that the amount by which the scaling exponents change when MSs are removed varies across participants, resulting in almost uncorrelated as well as antipersistent behaviour at the long time scale. This finding lends support to the fact that human observers are implementing drift and MSs in different ways to achieve accurate fixation over a long time. Consequently, an interaction of drift and MSs is likely to exist.

The second result we obtained is that the eyes tend to show oscillatory behaviour during drift movements, which manifests itself in oscillations of the MSD and DACF. This oscillatory behaviour is typical for systems with time-delayed feedback control^59,60,62^. It is commonly observed in the field of neurobiology and biological psychology^45,57,63,64,71^, as it takes a finite time to transmit and process signals within circuits of sensory, motor and processing units. Thus, our results provide evidence that drift movements are controlled by a time-delayed feedback rather than being a product of oculomotor noise. Due to the prolonged fixation duration and the large amount of trials, we were able to compute reliable estimates of the MSD and DACF for each of the 17 participants in this study. Interestingly, we found that the shape of the oscillations of the horizontal and vertical DACF vigorously varies across the participants. Nevertheless all participants have in common that the horizontal DACF reaches the first maximum at a larger time lag than the vertical one, thus indicating that the oscillatory behaviour of horizontal drift movements tends towards lower frequencies. These findings suggest that the way how subjects fixate a target differs not only with respect to MSs but also with respect to the oscillatory motions during drift. Therefore, it can be concluded that the hypothesised feedback control of drift movements is influenced by different factors which vary across human observers.

The functional role and control of drift movements in FEM is still under debate^1,19,41,42^. In a review of fixation experiments, Kowler and Steinman^19^ pointed out that about 85% of the tested subjects were capable of effective drift control, i.e., maintaining fixation in the absence of MSs, and thus suggested that a control system for drift movements is likely to exist. However, it is unclear where exactly in the brain such a control is implemented and how it works. On the basis of the time lags of the first maximum of the DACFs and the assumption that the period of oscillations is two to four times the delay time^63–65^, we estimated an approximate range for the time delay of about 20 ms to 70 ms. Hence, an involvement of cortical areas in the feedback control can be ruled out, as this would imply delays of an order of magnitude higher. A brain area which is compatible with this delay range and further likely to be involved in the control of drift movements is the superior colliculus, a retinotopic structure in the brainstem. Recent studies support that the superior colliculus is not only involved in the control and generation of saccadic eye movements^38,39^ but also in that of MSs^35,36^. This motivates to consider a further involvement of the superior colliculus in drift control^41,42^ which is also supported by recent findings^58,72^ stating that drift movements can be evoked by electrical stimulations of cells in the superior colliculus.

The SAW model of FEM proposed by Engbert *et al*.^44^ suggests a common control mechanism of drift movement and MSs, which is likely to be implemented in the superior colliculus. The model was motivated by the characteristic transition from persistence to antipersistence in the MSD, which it is able to reproduce. However, we also found that the SAW model does not reproduce the prominent oscillations in both the MSD and the DACF. Our third main result is that an implementation of a time-delayed position for the state readout in the SAW model is essential to generate oscillations of the DACF and MSD and more truthfully describes the experimental results. The simulated data of the DR-SAW and DAR-SAW model yields distinct, reasonable oscillations of the DACF for delays in the range of 20 ms to 36 ms, which is consistent with the delay range we estimated from the experimental data. Furthermore, this range is of the same magnitude as the signal transmission times for a retinal input transmitted to the superior colliculus (40 ms)^67^ and for motor commands sent by the superior colliculus to the eye muscles (20 ms)^39^. This points out that our implementation of a time delay is indeed consistent with the neurophysiological framework of the SAW model.

**Figure 8:**
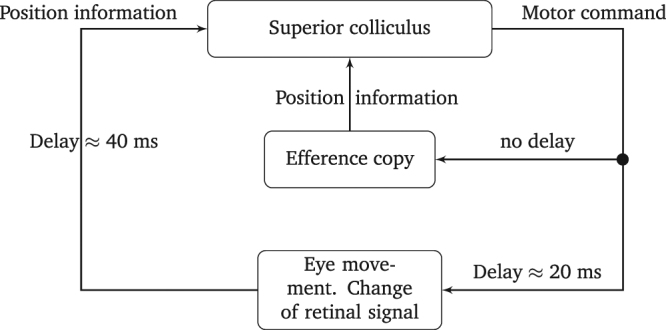
Scheme of a possible feedback loop for the control of drift movements. The values for the delays are taken from references^39,67^.

The lattice in the SAW model neurophysiologically represents the retinotopic map in the superior colliculus. Thus, the DAR-SAW and the DR-SAW suggest that the position of the eyes serves as a feedback signal and the question arises how the superior colliculus obtains this information. In general there are two ways in which the superior colliculus could gather information about the position of the eye, see Fig. 8. First, a visual stimulus could serve to estimate the position of fovea relative to the retinal image of the stimulus, using the retinotopic map in the superficial layer of the superior colliculus. Second, an efference copy^68,69^ of the generated motor commands, which innervate the extra-ocular muscles, could be integrated to predict the position of the eyes. Recent studies indeed provide evidence that the superior colliculus participates in an efference copy based feedback control of saccadic eye movements^58,73^. A motor command based position prediction of the eyes is possible since the muscles solely move the eye balls, which have a defined mass, and do not need to react to external, varying forces, e.g., like arm muscles during carrying a load. However, studies suggest that the integration of efference copy in the control of eye movements operates without delays^68,69^. Thus, a possible feedback control of drift movements is unlikely to be solely based on efference copy. An operative scheme of a feedback loop involving both types of position information is proposed in Fig. 8. Note that Skavenksi and Steinman^74^ suggest that retinal as well as extra-retinal control of the eye position is integrated during fixation, where the extra-retinal position control is mainly accomplished by saccades. Furthermore, a study of Soetedjo *et al*.^73^ provides evidence that the superior colliculus is involved in efference copy based feedback control of saccades. These findings motivate the use of the current position of the walker for the decision of MS triggering in the D-SAW models.

Considering these arguments, the DR-SAW model which integrates a delay of the position for the state readout but not for the activation setting could be an example for a separated integration of retinal and extra-retinal position information. However, it seems implausible that the state readout and the activation setting use separate feedback signals of the eye position. Nevertheless, a combined integration of retinal and motor signals for the control of drift is conceivable^74,75^. Taken all together we thus favour the DAR-SAW over the DR-SAW model.

To get a better understanding of the possible retinal control it could be useful to investigate the influence of different visual stimuli on the behaviour of the DACF, e.g., a lattice of black squares instead of a single black square or a fractal structure as investigated in different work^76^. In this context it is also of interest if the frequency of the eye oscillations correlate with the spatial frequency content of the presented images. This was hypothesised by Ahissar *et al*.^32^ who proposed a decoding scheme for visual signals which are temporally encoded by the retinal image motion of FEM. In this model high-acuity spatial resolution is achieved by integrating time delays of activity onsets in aligned clusters of horizontal and vertical elongated receptor cells during oscillatory cycles of FEM. The resolution of spatial details depends on the frequencies of the eye movements. Thus, Ahissar *et al*. suggested that if FEM are centrally controlled, subjects should adapt the frequencies of their FEM to the spatial frequency content of the presented image. This is an interesting indication of a possible functional role of feedback controlled FEM which could be further investigated with the methods presented here.

Furthermore, we addressed the problem to create a different behaviour of the horizontal and vertical component of drift movements in the DAR-SAW and DR-SAW as we observed for the experimental data. It turned out that different slope parameters of the quadratic potential for the horizontal and vertical direction yield a different shape of the horizontal and vertical DACF, which is indeed similar to that found in the experimental data. In general the potential slope in the SAW model is related to the fixation deviation, i.e., how good a subject can fixate a target. A variation of fixation deviation could be due to an involvement of top-down control in the fixation task, i.e., the conscious execution of a task, involving top level brain structures. Such a top-down control is likely to vary between the participants and thus could explain the diverse shapes of the DACF.

In summary it can be said that both SAW-type models are superior to the NDF model, since the SAW models generate both slow drift and microsaccades. The DR-SAW and the DAR-SAW are superior to the original SAW model, which could not reproduce the prominent oscillations of the MSD and DACF. However, for both DR-SAW and DAR-SAW models, the number of triggered MSs in a simulation trial shows large deviations compared to the SAW model and most MSs are triggered at marginal lattice sites with corresponding landing sites around the origin. This could be explained by the time-delayed state readout which enables the walker to visit lattice sites with high values of the potential more frequently while the activation at the lattice sites around the origin has enough time to decrease to a global minimum. Moreover it is important to check if other statistical properties of the SAW model are maintained like the exponential distribution of inter-microsaccadic intervals and the dynamical interaction of drift and MSs, i.e., the occurrence of slower drift prior to MSs. Therefore, some further refinements of the models are needed to reproduce the rich statistical-dynamical structure in experimental data. As a part of this, the special topography of the superior colliculus mapping, i.e. logarithimic scaling and the recently proposed different representation of the lower and upper visual field^70^ should be taken into account as well, which we did not consider in our current models.

## Methods

### Data acquisition

The binocular eye movement data was recorded by Engbert and Mergenthaler^77^ using an EyeLink II (SR Research, Osgoode, ON, Canada) eye tracker with a sampling frequency of 500 Hz and an instrument spatial resolution of 0.01°. To reduce head movements of participants a chin rest was used for stabilisation.

In the experiment 17 participants (students of the University of Potsdam) were asked to keep their eyes on a black square (3 × 3 px, spatial extent of 0.12°) displayed on a computer screen with white background. Each participant performed about 30 trials with a trial duration of 20 s. The data of a trial consists of a trajectory, {**x**_*i*_} = (**x**_1_, **x**_2_, …, **x**_*N*_), for each eye with horizontal and vertical eye positions, **x**_*i*_ = (*x*_hor_, *x*_ver_), measured in degrees and a sample size of *N* = 10,000.

In order to minimise the loss of trials, participants were asked to prevent eye blinks during trials. If missing data samples were detected (by an implemented automatic screening) the trial was aborted and then restarted. Due to the unnatural long fixation duration photographs were presented (10 s each) between trials to enable the participants to produce inspection saccades and eye blinks. All participants received a payment of 5 €.

Due to measurement errors and large saccades performed by the participants, 37 of overall 506 trials had to be discarded. These erroneous trials appear as outliers in the plot of the MSD. The distribution of discarded trials among the participants is depicted in Table 3.

**Table 3:** Distribution of erroneous trials among participants.

**Participant No**.	*1*	*2*	*5*	*9*	*10*	*13*	*14*	*15*	*16*
**Count of Err**. **Trials**	2	2	9	2	16	1	2	2	1

### Preprocessing

#### Smoothing

Due to measurement noise and the finite spatial resolution of the eye tracker, the recorded trajectories do not represent the exact movements of the eye. Thus a smoothing is applied to the data prior to the analysis. First the velocity is estimated,

5 $${{\bf{v}}}_{i}=\tfrac{{{\bf{x}}}_{i+2}+{{\bf{x}}}_{i+1}-{{\bf{x}}}_{i-1}-{{\bf{x}}}_{i-2}}{6{\rm{\Delta }}t},\,{\rm{for}}\,i=3\ldots N-2\,{\rm{and}}\,{{\bf{v}}}_{i}=\tfrac{{{\bf{x}}}_{i+1}-{{\bf{x}}}_{i-1}}{2{\rm{\Delta }}t},\,{\rm{for}}\,i\in \{2,N-1\}$$

using a weighted moving-average over five successive data samples. Here, Δ*t* = 2 ms according to the sampling frequency of the eye movement data. Note that the first and last velocity sample is estimated symmetrically. Next, a cumulative summation of the estimated velocity samples **v**_*i*_,

6 $${{\bf{x}}}_{i}^{s}={{\bf{x}}}_{1}+{\rm{\Delta }}t\,\sum _{j=2}^{i-1}\,{{\bf{v}}}_{j},$$

is performed to obtain the smoothed trajectory $\{{{\bf{x}}}_{i}^{s}\}$.

#### Detection of microsaccades

Microsaccades are detected using an elliptical velocity threshold,

7 $${(\frac{{v}_{x,i}}{{\eta }_{x}})}^{2}+{(\frac{{v}_{y,i}}{{\eta }_{y}})}^{2} > 1,\,{\rm{with}}\,{\eta }_{x,y}=\lambda {\sigma }_{x,y}=\lambda \sqrt{\langle \,{{v}_{x,y}}^{2}\rangle -{\langle {v}_{x,y}\rangle }^{2}},$$

whose major and minor axes are determined by the variance *σ* of the horizontal and vertical velocity samples. The parameter *λ* can be used to adjust the threshold to experimental conditions. Here we used a fixed value of *λ* = 5. Note that we used the median for the calculation of the variance *σ*. Thus the threshold is more resilient to large velocity values, i.e, more sensitive to smaller MSs.

A microsaccade is defined as a succession of at least three velocity samples (equivalent to 6 ms) falling outside of the elliptical velocity threshold. To reduce noise in MS detection, the fact that MS are binocular is utilised. A binocular MS is detected if there is a temporal overlap of monocular MSs (left and right eye) of at least one velocity sample, which can be expressed by the criterion, *r*_end_ > *l*_onset_ *and r*_onset_ < *l*_end_, for the MS *onset* and *end* of the left (*l*) and right (*r*) eye. Microsaccades which do not match this criterion are rejected.

Trajectories with removed MSs are obtained by applying the cumulative summation to the velocity time series, see Eq. (6), after the corresponding velocity samples have been removed.

For further reading on the method of smoothing and MS detection, see ref.^78^. The R scripts for the smoothing and MS detection are available on-line in the Microsaccade Toolbox^79^.

### Correlation analyses

For the correlation analyses we used the mean squared displacement (MSD) and the displacement autocorrelation function (DACF). Due to the limited amount of trials, available for each of the participants, we applied a time average^53^ instead of the common ensemble average, for the estimation of the MSD and DACF, see Eqs (8) and (9), respectively,

8 $${\rm{MSD}}(l)=\frac{1}{N-l}\,\sum _{i=1}^{N-l}\,{({{\bf{x}}}_{i+l}-{{\bf{x}}}_{i})}^{2},$$

9 $$\overline{{\rm{DACF}}}(l)=\frac{1}{N-l-k}\,\sum _{i=1}^{N-l-k}\,\frac{({{\bf{x}}}_{i+l+k}-{{\bf{x}}}_{i+l})\cdot ({{\bf{x}}}_{i+k}-{{\bf{x}}}_{i})}{{k}^{2}},$$

where *N* denotes the sample size of the trajectory. The time-averaging allows us to obtain more stable estimations of the MSD and DACF for each participant, which we furthermore averaged over the ensemble of participants. Both estimators were implemented in R and Matlab. Note that the normalised DACF, ${\rm{DACF}}(l)=\overline{{\rm{DACF}}}(l)/\overline{{\rm{DACF}}}\mathrm{(0)}$, with respect to *l* = 0, is used for the plots. The estimation for the MSD, see Eq. (8), was taken from Collins and DeLuca^61^. The implementation of the DACF estimator is straight forward according to the MSD estimator. For the computation of the DACF we used a fixed time interval *k* = 25, which is equal to 50 ms. Note that we estimated the scaling exponent *α* of the MSD with a linear regression of the logarithms of the MSD in the range of *τ* ∈ (2 ms − 12 ms) and *τ* ∈ (120 ms − 1200 ms) for the short and long time scale, respectively.

### Computational models of FEM

#### Non-linear delayed feedback model (NDF)

The NDF model, see Eq. (10), proposed by Mergenthaler *et al*.^45^, is basically a first-order autoregressive map of the activity of excitatory burst neurons, *w*_*i*_, with an additional time-delayed, non-linear, negative feedback term, −*λ* tanh(*εw*_*i*−*τ*_). The mapping of the activity *w*_*i*_, which is related to the eye velocity, is driven by Gaussian white noise *ξ*_*i*_, (〈*ξ*_*i*_〉 = 0, 〈*ξ*_*i*_ *ξ*_*j*_〉 = *σ* ^2^ *δ*_*ij*_), representing the baseline activity of the excitatory burst neurons. The autoregressive term refers to the inertia of the eye balls. The position of the eye, *x*_*i*+1_, is determined by the sum of the previous position *x*_*i*_, the current activity of excitatory burst neurons *w*_*i*+1_ and a Gaussian white noise term *η*_*i*_, (〈*η*_*i*_〉 = 0, 〈*η*_*i*_ *η*_*j*_〉 = *ρ* ^2^ *δ*_*ij*_), representing noise in the activity of tonic units, i.e. eye-position-related neurons. Thus,

10 $$\begin{array}{rcl}{w}_{i+1} & = & \mathrm{(1}-\gamma )\,{w}_{i}+{\xi }_{i}-\lambda \,\tanh (\varepsilon {w}_{i-\tau }),\\ {x}_{i+1} & = & {x}_{i}+{w}_{i+1}+{\eta }_{i}\mathrm{.}\end{array}$$

For the simulations we used the following parameter set: *γ* = 0.25, *λ* = 0.15, *ε* = 1.1, *σ* = 0.075, *ρ* = 0.35, *τ*_hor_ = 35 and *τ*_ver_ = 20, which turned out to yield the best match of simulated and experimental data^45^.

#### Fading self-avoiding walk model (SAW)

The fading self-avoiding walk (SAW) proposed by Engbert *et al*.^44^ is set up on a *L* × *L* linearly scaled square lattice. During each iteration, i.e. time step, the walker locally increases an activation field *h* at his current position (*i*, *j*),

11 $${h}_{ij}\to {h}_{ij}+\mathrm{1,}$$

while the activation field decays globally with each iteration,

12 $${h}_{kl}=\mathrm{(1}-\varepsilon )\,{h}_{kl},\quad {\rm{with}}\quad (k,l)\in L\times L,$$

where *ε* is the relaxation rate. The self-avoidance is implemented by the principle that the walker will always step on one of his four neighbouring lattice sites, which exhibits the lowest value of the sum of the activation *h* and the quadratic potential *u*,

13 $$(i^{\prime} ,j^{\prime} )=\mathop{{\rm{\arg }}\,{\rm{\min }}}\limits_{(k,l)\in N(i,j)}\,\{{h}_{kl}+u(k,l)\},\,{\rm{with}}\,N(i,j)=\{(k,l)|k=i\pm 1,\,{\rm{and}}\,l=j\pm 1\}.$$

When two or more neighbouring lattice sites exhibit the same value for the sum of *h* and *u*, one of them is determined with equal probability as the next position. The quadratic potential,

14 $$u(i,j)=\lambda L\,({(\frac{i-{i}_{0}}{{i}_{0}})}^{2}+{(\frac{j-{j}_{0}}{{j}_{0}})}^{2}),$$

serves as a restoring force, which tends to drag the walker towards the centre of the lattice and thus keeps the walker within the bounds of the lattice. When the walker steps on a lattice site with an activation above a defined threshold, *h*_*c*_, an MS is triggered towards the lattice site (*i*′, *j*′) with the global minimum of the sum of the activation *h*, the quadratic potential *u* and the microsaccadic potential *u*_1_,

15 $$(i^{\prime} ,j^{\prime} )=\mathop{{\rm{\arg }}\,{\rm{\min }}}\limits_{(k,l)\in L\times L}\,\{{h}_{kl}+u(k,l)+{u}_{1}(k,l)\},\,{\rm{with}}\,{u}_{1}(i,j)=\chi L\,{(\frac{i-{i}_{0}}{{i}_{0}})}^{2}\,{(\frac{j-{j}_{0}}{{j}_{0}})}^{2}.$$

The potential *u*_1_ whereby ensures that MSs occur predominantly in the horizontal and vertical direction.

With the following set of parameters: *λ* = 1, *χ* = 2, *ε* = 10^−3^, *h*_*c*_ = 7.9, and *L* = 51, the simulated data of the SAW model matches the experimental data best^44^. Note that the simulation starts with a so called burn-in of about 10^4^ iterations, using an initial activation field of random numbers, drawn from a uniform distribution in the interval (0, 1). Furthermore, the centre of the lattice is used as the initial position for the burn-in. The actual simulation, then uses the final position and the activation field of the burn-in as the initial conditions, while the trajectory of the burn-in is rejected.

### Data availability statement

The datasets analysed during the current study are available from the corresponding author on reasonable request.
